# Maternal administration of cannabidiol promotes an anti-inflammatory effect on the intestinal wall in a gastroschisis rat model

**DOI:** 10.1590/1414-431X20177132

**Published:** 2018-03-15

**Authors:** G.H. Callejas, R.L. Figueira, F.L.L. Gonçalves, F.A.P. Volpe, A.W. Zuardi, J.A. Crippa, J.E. Hallak, L. Sbragia

**Affiliations:** 1 Laboratório de Cirurgia Experimental Fetal e Neonatal “Michael Harrison” Divisão de Cirurgia Pediátrica, Departamento de Cirurgia e Anatomia Faculdade de Medicina de Ribeirão Preto, Universidade de São Paulo, Ribeirão Preto, SP, Brasil; 2 Departmento de Neurociências e Comportamento, Faculdade de Medicina de Ribeirão Preto, Universidade de São Paulo, Ribeirão Preto, SP, Brasil

**Keywords:** Gastroschisis, Experimental model, Prenatal treatment, Cannabidiol

## Abstract

Gastroschisis (GS) is an abdominal wall defect that results in histological and morphological changes leading to intestinal motility perturbation and impaired absorption of nutrients. Due to its anti-inflammatory, antioxidant, and neuroprotective effects, cannabidiol (CBD) has been used as a therapeutic agent in many diseases. Our aim was to test the effect of maternal CBD in the intestine of an experimental model of GS. Pregnant rats were treated over 3 days with CBD (30 mg/kg) after the surgical induction of GS (day 18.5 of gestation) and compared to controls. Fetuses were divided into 4 groups: 1) control (C); 2) C+CBD (CCBD); 3) gastroschisis (G), and 4) G+CBD (GCBD). On day 21.5 of gestation, the fetuses were harvested and evaluated for: a) body weight (BW), intestinal weight (IW), and IW/BW ratio; b) histometric analysis of the intestinal wall; c) immunohistochemically analysis of inflammation (iNOS) and nitrite/nitrate level. BW: GCBD was lower than CCBD (P<0.005), IW and IW/BW ratio: GCBD was smaller than G (P<0.005), GCBD presented lower thickness in all parameters compared to G (P<0.005), iNOS and nitrite/nitrate were lower concentration in GCBD than to G (P<0.005). Maternal use of CBD had a beneficial effect on the intestinal loops of GS with decreased nitrite/nitrate and iNOS expression.

## Introduction

Gastroschisis (GS) is a congenital abdominal wall defect characterized by a small hole, generally located to the right of the navel, resulting in the herniation and permanent exposure of the bowel loops to amniotic fluid and its components during pregnancy (1). This exposure promotes morphological and histological changes to the intestinal wall, such as reduction of intestine length, thickening of intestinal layers, and disturbance of neuronal cells maturation, leading to intestinal hypomotility and impaired absorption of nutrients, which in turn raises the occurrence of post-operatory complications, elevating the morbidity, mortality, and time of stay treatment cost in the neonatal period (2,3).

Simple GS may have a survival rate close to 100%. On the other hand, association with malrotation, infarction, atresia, perforation, and stenosis has a mortality rate of 28%, besides higher risk of long-term complications such as short gut syndrome (1). Molecular mechanisms involved in GS have not been completely unraveled. However, it is known that its inflammatory process causes histological and physiological changes associated with high local concentration of nitric oxide (NO) and nitric oxide synthases (NOS) (4).

NO is a free radical gas that has numerous molecular targets and a short half-life in biological systems. As a method to indirectly measure the levels of NO, the concentration of nitrite, a stable product of NO formation, in blood and tissue has been used as routine (5,6).

In normal conditions, NO and NOS are expressed at low levels. NO promotes smooth muscle relaxation and it is involved with epithelial permeability, whereas at high levels NO can affect the gut barrier permeability leading to increased bacterial translocation and epithelial damage. NO production is mediated mainly by two NOS, eNOS (endothelial) and iNOS (induced) through catalysis. eNOS is constitutive, while iNOS is upregulated during inflammatory processes increasing NO production (6–8).

Prevention or reduction of the inflammatory intestinal fetal lesion has been a recent target of treatment strategy in experimental GS. Maternal administration of corticosteroids showed, beside the anti-inflammatory action, favorable effects upon fetal pulmonary and intestinal development (2,3,9,10). However, this strategy may lead to deleterious effects such as increased maternal infection (11), brain growth deficits, periventricular leukomalacia, poor attention and cognitive performance (12), and low neuropsychiatric functioning (13). Thus, there is a clear need to explore new pharmacological possibilities for antenatal management.

Cannabidiol (CBD) possesses anti-inflammatory, anti-oxidant and neuroprotective effects with a wide range of possible therapeutic effects in diseases such as Parkinson's, Alzheimer's, brain ischemia, diabetes, nausea, cancer, and rheumatoid arthritis (14). This cannabinoid has shown a favorable safety profile in humans (15). CBD can modulate tumor necrosis factor *in vitro* and suppress the production of chemokines by human B cells in a model of rheumatoid arthritis in synovial cells isolated from treated knees (16). In acute inflammation and experimental chronic pain, it has had a beneficial effect upon edema and hyperalgesia (17,18). Considering the bowel loops inflammation and the immaturity of the myenteric plexus that occur in fetal GS (19,20), our aim was to evaluate the effect of maternal administration of CBD in an experimental rat model of the disease.

## Material and Methods

This study was approved by the Ethics Committee in Animal Experimentation of Faculdade de Medicina de Ribeirão Preto, Universidade de São Paulo, Ribeirão Preto, SP, Brazil (#215/2014).

### Animals

Female Sprague*-*Dawley rats were submitted to mating. A male/female pair was maintained together overnight. The following morning, a vaginal swab was performed and the presence of sperm marked day zero of pregnancy (time of gestation, 22 days).

### Groups

A total of 10 pregnant rats were operated, resulting in 40 fetuses that were subdivided in 4 groups (n=10 per group): 1) control (C); 2) control+CDB (CCBD); 3) gastroschisis (G), and 4) gastroschisis+CBD (GCBD). The maternal administration of CBD was 30 mg/kg via intraperitoneal (*ip*) injection, once per day for 3 days (from surgical day 18.5 until harvest day 21.5).

### Gastroschisis surgical procedure

Surgery was performed at 18.5 days of gestation. Pregnant rats were anesthetized with 50 mg/mL ketamine (Ketamina¯, Pfizer, Brazil) and 10 mg/mL xylazine (Rompum¯, Bayer, Brazil). The abdominal cavity was opened by median laparotomy in 2 layers. Fetuses were counted from the uterine isthmus, and GS was created on the second fetus of each horn according to the technique described by Correia-Pinto et al. (21). The lower body of the fetus was exposed and a right para-umbilical laparotomy with standardized extension of 5 mm was performed, opening the fetal abdominal cavity with caution not to damage the umbilical vessels and liver. The bowel loops were gently exposed by the delicate compression of the fetal abdomen with cotton swabs. After creating the defect, the fetus was carefully placed back into the uterine cavity, and a previously placed purse string suture closed the uterus.

### CBD Preparation

CBD was provided in powder form with 99.9% purity (BSPG-Pharm, UK). The drug was suspended in polyoxyethylenesorbitan monooleate (Tween 80) 2% saline, at a concentration of 30 mg/kg. The concentration was based on previous studies in rat neonates and adapted to this model (22). CBD was administrated in the pregnant rat by *ip* injection on 18.5, 19.5, and 20.5 days of gestation.

### Harvest

Pregnant rats were submitted to harvesting on day 21.5. Fetuses were removed, weighed, and dissected to harvest the ileum. The intestinal segment was removed from ileum until the proximal colon, approximately 2 cm. The tissue was then fixed in 10% formalin solution for immunohistochemistry and histologic analysis, and frozen for nitrite/nitrate analysis.

### Morphological and histometric analysis

Body weight (BW), intestinal weight (IW), and the IW/BW ratio were analyzed (n=10). For weight analysis, the whole intestine was considered (wet weight). The intestinal histology samples from ileum were stained by Masson’s trichrome and photographed using the Nikon Eclipse E200 80i photomicroscope (Nikon, Japan), with a 400× magnification for measurement of the intestinal wall layers. The images were digitized, allowing measurement of the layers serosa (SE), longitudinal muscle (LM), circular muscle (CM), submucosal mucous membrane (SM), and total wall (TL) using Image Pro Plus 6.0 software (Media Cybernetics Inc., USA). Twelve fields per slide were analyzed in a total of 4 slides per group.

### iNOS Immunohistochemical analysis

Twelve fields per slide and four slides per group were analyzed by three blind investigators. The immunohistochemistry score was graded according to the intensity of staining from 0 to 4: 0=negative, 1=weak staining, 2=moderate staining, 3=strong staining, 4=very strong staining (23).

The slides were deparaffinized in xylol and dehydrated with ethanol. After these steps, blocking of endogen peroxidases were prepared by incubation of the slides in H_2_O_2_ (30%) and 3% methanol for 10 min. For antigenic exposure, the slides were placed in 50 mM Tris-HCl buffer solution, pH 9.5 (containing 5% urea) and in an Optisteam Plus steamer, model 652, (Krups North America, USA) for 40 min, then cooled in an ice water bath for 15 min, and lastly washed in distilled water. Blocking was performed with 10% goat serum in phosphate buffered saline (PBS, Na_2_HPO_4_ 20 mM + NaCl 0.45 M, pH 7.4) for 30 min in a humidity chamber. The slides were incubated overnight at 4°C with the primary antibody (rabbit anti-iNOS sc-651 Santa Cruz Biotechnology, USA) diluted 1:200 in PBS with 3% bovine serum albumin (BSA). After removal of the primary antibody with PBST 2% solution, a Polymer Detection Kit (MACH 4 Universal HRP-Polymer M4U534, Biocare Medical, USA) was added for 30 min. The printing was performed with a solution of 3,3′-diaminobenzidine-tetra-hidrocloret (Sigma, USA) and 0.03% hydrogen peroxide in triphosphate buffered saline (TBS) 0.05 M, pH 7.6, for 5 min. Lastly, the slides were counter-stained with Harris hematoxylin, washed in running water, dehydrated by a series of ascending concentrations of alcohol and xylol baths, and cover slipped with Permount¯ (Fisher Scientific, USA).

### Sample preparation for Griess reaction

Each 100 µg of intestine was homogenized in 400 µL of buffer containing 10 mM EDTA, 100 mM Trizma base, 10 mM sodium pyrophosphate, 100 mM sodium fluoride, 10 mM sodium orthovanadate, 0.1 mg/mL aprotinin, 2 mM phenylmethylsulfonyl fluoride, and distilled water then centrifuged in a Mikro 200R centrifuge (Hettich, Germany) at 12,000 rpm at 4°C for 30 min; the supernatant was aliquoted for analysis. To avoid the process of tyrosine nitration and S-nitrosocysteine formation observed in biological samples submitted to slow freezing, we rapidly froze our samples in liquid nitrogen (11).

### Determination of nitrite/nitrate concentration by Griess reaction

The concentration of nitrite/nitrate (NO_2_/NO_3_) was performed from the terminal ileum homogenate by the Griess reaction after enzymatic reduction of nitrate to nitrite by nitrate reductase enzyme (Sigma, USA) in a reducing solution containing 0.5 M phosphate buffer, pH 7.5, and NADPH. For this reaction, a 96-well flat bottom (USA Corning, USA) plate was used in which were deposited 40 µL of the reducing solution and 40 µL of each sample in triplicate, and maintained at 37°C for 12 h. Then, nitrite was detected through the addition of 20 µL of Griess reagent. After 10 min, the absorbance was measured at 540 nm using an automatic microplate reader (Molecular Devices, Spectra Max 250, USA). Nitrite concentrations were calculated based on the standard curve of NaNO_2_ and data are reported as µM/µg intestinal tissue (24).

### Statistical analysis

For morphological, histological, and nitrite/nitrate data analysis, ANOVA and Tukey’s post-test were used. For immunohistochemistry concentration data, the Kruskal Wallis with Dunn’s post-test were used. Significance was considered when P<0.05. The software used was GraphPad Prism 6.0 (GraphPad Software Inc., USA). Results are reported as means±SD, and median and interquartile range (IQR) for immunohistochemistry analysis.

## Results

### Morphological and histometric analysis

**Figure 1.**
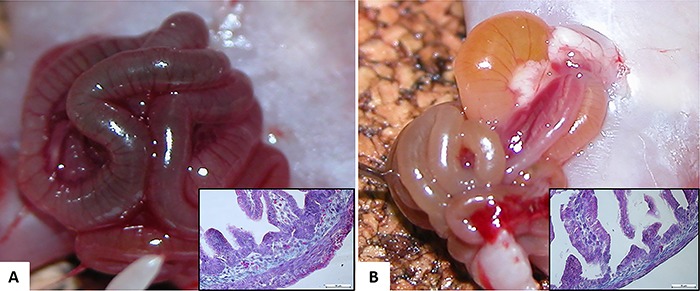
*A*, Macroscopic view of the intestinal evisceration of the gastroschisis (G) group (time of harvest). Inset: histological representation of ileum specimen stained with H&E of the G group. *B*, Macroscopic view of the intestinal evisceration of the gastroschisis+cannabidiol (GCBD) group (time of harvest). Inset: histological representation of the ileum specimen stained with H&E of the GCBD group. Note the different aspect and thickness of the bowel loop macroscopically and microscopically between the 2 groups. Masson's trichrome staining from ileum segment. Magnification: 100×. Scale bar: 10 μm.

Figure 1 shows macroscopical and microscopical differences of the bowel loop between G and GCBD groups.

**Figure 2.**
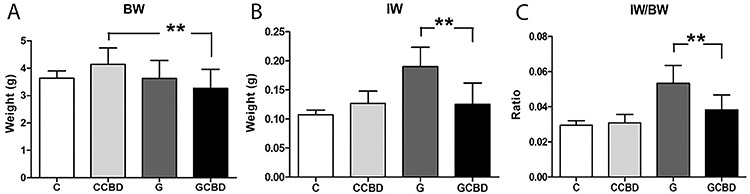
Morphological analysis. Data are reported as means±SD. *A*, body weight (BW; g). *B*, intestinal weight (IW, g). *C*, Intestinal weight/body weight ratio (IW/BW). C: control; CCBD: control+cannabidiol; G: gastroschisis; GCBD: gastroschisis+cannabidiol. **P<0.005 (ANOVA).

A significant difference was found for BW between CCBD *vs* GCBD (4.142±0.598 *vs* 3.270±0.683 g, respectively P<0.005). For IW analysis, the GCBD group showed reduced values (0.124±0.037 g) compared to the G group (0.190±0.033 g, P<0.005) and similar to the other groups C and CCBD (0.107±0.008 and 0.126±0.021 g, respectively, P>0.05). The GCBD group showed lower IW/BW ratio (0.038±0.008) compared to G group (0.053±0.010, P<0.005) and higher than the C group (0.029±0.002), and there was no difference to the CCBD group (0.030±0.004, P>0.05). Results are shown in Figure 2.

**Figure 3.**
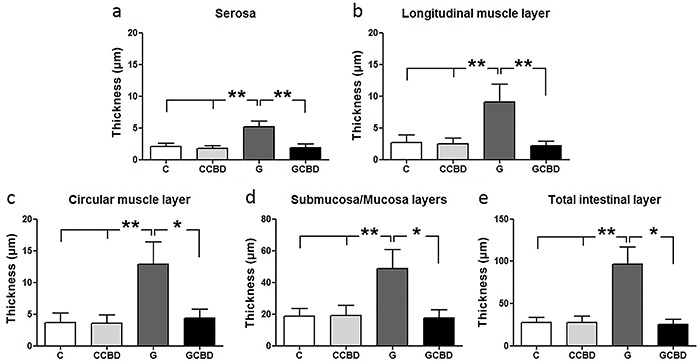
Histometric analysis of the intestinal layers. Data are reported as means±SD. C: Control; CCBD: Control + cannabidiol; G: Gastroschisis; GCBD: Gastroschisis + cannabidiol. *P<0.05; **P<0.005 (ANOVA).

In the histometric analysis of tissue layers, (SE, ML, CM, SM, and TL), the GCBD group presented reduced thickness compared to group G, and no difference compared to groups C and CBD in any of the layers (Figure 3).

### iNOS immunohistochemical analysis

**Figure 4.**
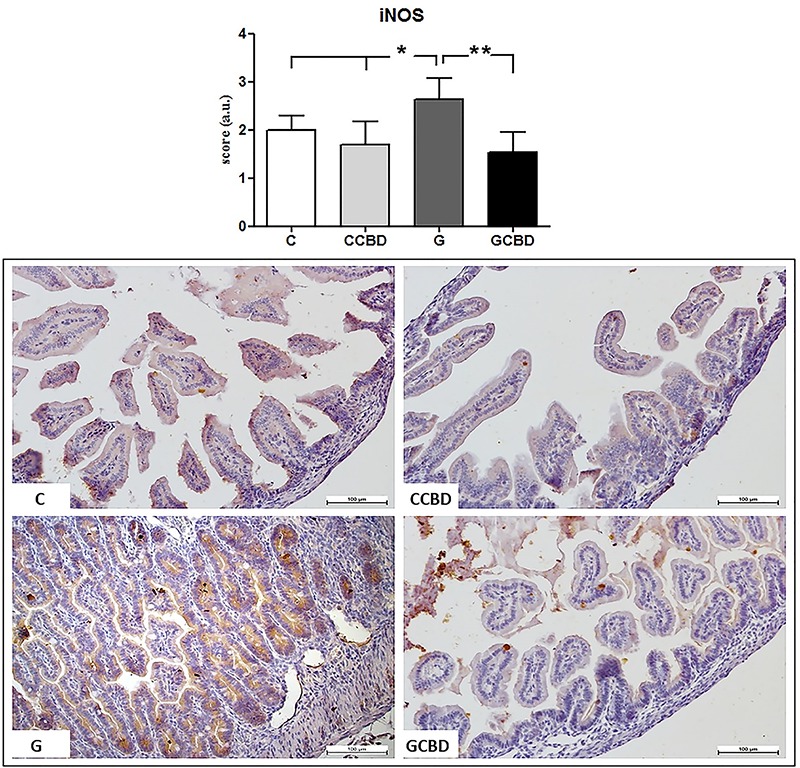
Immunohistochemistry score for iNOS expression and the histological transversal slides stained by immunohistochemistry. Data are reported as means±SD. Magnification 200×, bar=100 μm. C: control; CCBD: control+cannabidiol; G: gastroschisis; GCBD: gastroschisis+cannabidiol. *P<0.05; **P<0.005 (Kruskal Wallis).

The group GCBD showed reduced iNOS activity in comparison to the G group, and similar to the groups C and CCBD (Figure 4).

### Nitrite/nitrate concentration of the intestine tissue homogenate

**Figure 5.**
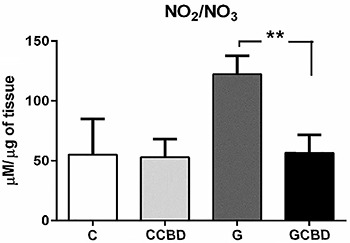
Nitrite/nitrate concentration in intestine tissue homogenate. Data are reported as means±SD C: control; CCBD: control+cannabidiol; G: gastroschisis; GCBD: gastrosquisis+cannabidiol. **P<0.005 (Kruskal Wallis).

The GCBD group showed reduction of NO_2_/NO_3_ concentration compared to group G and was similar to groups C and CCCB, (56.862±14.644 *vs* 122.377±15.463, P<0.005, 55.249±29.607 and 53.120±15.089, P>0.05, respectively, Figure 5).

## Discussion

GS is an abdominal wall defect that occurs in 1/4,000 live births and is increasing all over the world (25,26). Maternal therapy with dexamethasone in a GS rat model showed reversion of intrauterine growth restriction (IUGR), reduction of intestinal weight and mucosal thickness (3,5). Treatment with hydrogel associated to an NO donor covering the GS fetal bowel loops as well as low concentration release of NO in the amniotic fluid reduced morphological parameters and intestinal wall thickness (4,27).

Enteric glial cells actively mediate acute and chronic inflammation in the intestine promoting proliferation and release of neurotrophins, growth factors and pro-inflammatory chemokines that in turn may increase the immunological response, representing a very important link between the nervous and immune system in the intestine. CBD is an interesting compound due to its capacity to control reactive gliosis in the central nervous system, without psychotropic effects and safe toxicological profile (15,28,29).

In this study, we verified that maternal intraperitoneal administration of CBD had no effect on BW, which showed difference only between the group CCBD and GCBD, with the former presenting higher values. While the effect of CBD in IUGR in clinical and experimental fetuses is not well known, it has been demonstrated that longer exposure to CBF of the amniotic fluid worsens IUGR in an experimental model in rats (30), which might be linked to fetal alterations responsible for glucose and insulin receptors modification by fetal inflammation (31). Such modification could mean that CBD injection, even if intraperitoneal, in a control (healthy) organism in growth phase could have an effect on body mass gain, without the same effect seen in the GCBD group (treated disease). Wasserman et al. (32) verified, in C57bl/6j mice with ages between 5 and 11 weeks, that cannabinoid receptors CB1 and CB2 are specifically located in hypertrophic chondrocytes of the epiphyseal growth cartilage, which drives vertebrate growth. Additionally, blocking CB2 receptors slows skeleton elongation, which could explain body growth changes in the healthy newborns treated with CBD found in our research. It is important to highlight that the increase of BW in the CCBD group was only significant when compared to the GCBD group, and that, when compared to the C group, did not show differences in any of the parameters (BW, IW, IW/BW). At first, this could be an evidence that this treatment has no risk of impairment related to weight.

An increased IW and IW/BW ratio was observed in the G group compared to the other groups (C, CICBD, GCBD) and a reduction was observed in the GCBD group compared to G. Such findings might demonstrate that CBD use resulted in benefits to the GCBD group with a potential reversion of the inflammatory reaction caused by the exposure of the bowels to the amniotic fluid. Additionally, no significant difference was found between groups GCBD and C in any of the parameters. Therefore, the results of the morphological analysis demonstrated potential benefits induced by the treatment with CBD.

Furthermore, the histometric analysis showed a reduction of the intestinal layer thickness in the GCBD group compared to the G group and had similar results to the other control groups. The normality of this parameter of intestinal inflammation corroborates the hypothesis that CBD not only was effective in reducing the intestinal inflammation, but also did not cause any impairment to intestinal development.

The iNOS expression was reduced in group GCBD in relation to G group. The increased NOS expression in GS could mediate hypoperistalsis and malabsorption (33). In addition, a study showed that inhibition of NOS was correlated with reduction of intestinal thickness and improvement of bowel loops macroscopic appearance of GS in chick embryos (34). In previous studies, we had observed the reduction of iNOS in GS fetuses after treatment with a NO donor (20). In the literature, the inhibiting effect of CBD over iNOS has previously been described in an *in vivo* model of neuroinflammation; mice that suffered beta-amyloid protein-induced neurological inflammation received treatment with intraperitoneal CBD and showed reduction of iNOS expression in relation to the non-treated diseased group (35).

However, a study that evaluated the effect of CBD in a neuropathic pain model and sciatic nerve lesion did not show reduction of iNOS expression with CBD, suggesting that the relationship between CBD and NOS might show peculiarities in different tissues. Rats with inflamed paws or sciatic nerve lesion had an increase of iNOS, nNOS, and eNOS expression. Treatment with CBD only reduced the overexpression of eNOS in rats with inflamed paws, without reaching similar levels as the control group, while there was no significant reduction of iNOS and nNOS (17).

A diabetes neuropathy study in rats suggests that NOS could have an inhibitory effect over cannabinoid receptor agonists. According to the authors, the use of a specific nNOS inhibitor (7-Ni) and a relatively selective inhibitor of iNOS (L-NIL) showed a positive impact over the analgesic effect of two cannabinoid receptors agonists. Such finding suggests that CBD might not only modulate the expression and activity of NOS isoforms, but also have an inhibitory effect on the analgesia and anti-inflammatory pathways activated by cannabinoids agonists (36). In this study, we observed the reduction of nitrite/nitrate concentration in the GCBD group in comparison to the G group; values were similar to the groups C and CBD, which indirectly shows the reduction of NO concentration in the treated group in comparison to the diseased non-treated group. The NO reduction could be explained by the decrease of cytotoxic action mediated by macrophages and other immune cells, but further studies should be made to confirm this hypothesis.

CBD does not modify intestinal motility in control animals, but can inhibit the hypermotility associated to experimental ileitis in mice (37). Borrelli et al. (38) evaluated the CDB effect in experimental colitis in mice and verified a reduction of intestinal inflammation shown by the reduction of iNOS and the interleukins 1-beta and 10, as well as reduction of the intestinal mucous oxidative stress. Lin et al. evaluated the activity of the cannabinoid receptor G Protein-coupled Receptor 55 (GPR55) and its ligands O-1602 in the gut movement of rodents with lipopolysaccharide (LPS)-induced intestinal inflammatory disease and concluded that CBD selectively normalizes motility perturbation through potential mechanisms involving systemic anti-inflammatory effect and regulation of the myoelectrical activity in the intestine (39). De Filippis et al. (28) also confirmed in human biopsy samples and in mice with LPS-induced enteritis that CBD treatment resulted in reduction of iNOS and S100B.

We aimed to evaluate the CBD impact on intestinal inflammation promoted by amniotic fluid exposure. Our study had some limitations. Although the analyzed parameters were not direct indications of an inflammatory state, they have been previously studied and correlated with GS as a consequence of the inflammation and the cannabidiol treatment showed similar results in comparison to those observed in previous studies that aimed reduction of the inflammation of the intestine (3,9,10). Also, we focused on the intestine responses and thus did not discuss the cannabidiol effects in the brain or other organs, although we can assert that clinical and visible alterations were not observed in the rats.

There was no evidence in the analyzed parameters that the administration of CBD 30 mg/kg via *ip* in female pregnant rats have caused impairments to fetal development in the diseased (GCBD) or unaffected groups (CCBD). Although CBD treatment did not correlate with any changes between C and CCBD groups in the evaluated parameters, long term studies are necessary to demonstrate possible secondary effects of CBD.

Finally, the treatment with CDB of experimental GS in rats reduced the weight, thickness of intestinal layers, concentration of NO_2_/NO_3_, and expression of iNOS of the bowel loops showing an effective anti-inflammatory action and pharmacological application for pre-natal use. Further studies could clarify the interaction between NO and CBD pathways. In addition, investments for future studies translating the present findings to the clinical practice are desirable and suitable.

### Declaration of potential conflict of interest

A.W. Zuardi, J.E. Hallak, and J.A. Crippa are co-inventors (Mechoulam R, JAC, Guimaraes FS, AWZ, JEH, Breuer A) of the patent “Fluorinated CBD compounds”, compositions and uses thereof. Pub. No.: WO/2014/108899. International Application No.: PCT/IL2014/050023” Def. US no. Reg. 62193296; 29/07/2015; INPI on 19/08/2015 (BR1120150164927). The University of São Paulo has licensed the patent to Phytecs Pharm (USP Resolution No. 15.1.130002.1.1). The University of São Paulo has an agreement with Prati-Donaduzzi (Toledo, Brazil) to “develop a pharmaceutical product containing synthetic cannabidiol and prove its safety and therapeutic efficacy in the treatment of epilepsy, schizophrenia, Parkinson's disease, and anxiety disorders”. JAC and JEH have received travel support from and are medical advisors of BSPG-Pharm.
